# Tumor Necrosis Factor-****α**** as a Diagnostic Marker for Neonatal Sepsis: A Meta-Analysis

**DOI:** 10.1155/2014/471463

**Published:** 2014-02-11

**Authors:** Bokun Lv, Jie Huang, Haining Yuan, Wenying Yan, Guang Hu, Jian Wang

**Affiliations:** ^1^Systems Sepsis Team, Soochow University Affiliated Children's Hospital, Suzhou 215003, China; ^2^Center for Systems Biology, Soochow University, Suzhou 215006, China; ^3^Suzhou Zhengxing Translational Biomedical Informatics Ltd., Taicang 215400, China

## Abstract

Neonatal sepsis (NS) is an important cause of mortality in newborns and life-threatening disorder in infants. The meta-analysis was performed to investigate the diagnosis value of tumor necrosis factor-**α** (TNF-**α**) test in NS. Our collectible studies were searched from PUBMED, EMBASE, and the Cochrane Library between March 1994 and August 2013. Accordingly, 347 studies were collected totally, in which 15 articles and 23 trials were selected to study the NS in our meta-analysis. The TNF-**α** test showed moderate accuracy of the diagnosis of NS both in early-onset neonatal sepsis (sensitivity = 0.66, specificity = 0.76, *Q∗* = 0.74) and in late-onset neonatal sepsis (sensitivity = 0.68, specificity = 0.89, *Q∗* = 0.87). We also found the northern hemisphere group in the test has higher sensitivity (0.84) and specificity (0.83). A diagnostic OR analysis found that the study population may be the major reason for the heterogeneity. Accordingly, we suggest that TNF-**α** is also a valuable marker in the diagnosis of NS.

## 1. Introduction

Neonatal sepsis (NS), as one of the major causes, leads to neonatal mortality and morbidity, especially in neonates born preterm [1]. Early diagnosis and management of the newborn infant with NS play key roles in preventing severe and life-threatening complications [2]. During the first hours of life, reliable infection markers are absent in NS. Therefore, neonatologists often begin early antibiotic treatment in newborn infants with risk factors for infection, exposing many neonates to unnecessary treatments. Due to the limitation of the treatment strategy in early diagnosis of sepsis, the isolation of causative organisms from microbiological cultures takes up to 72 h, which cannot be used to identify most infected infants [3].

Procalcitonin (PCT), a 116-amino acid peptide considered as a precursor in calcium homeostasis, has been proved to be a valuable marker for distinguishing sepsis from other noninfectious disease [4, 5]. C-reactive protein (CRP), another biomarker, is a protein discovered in the blood and whose levels will rise after inflammation [6–8]. To date, CRP and PCT have been proposed for inclusion as two mostly used diagnostic markers in the international definition of sepsis [9, 10]. Recently, many studies have found that some new markers also play important roles in the diagnosis of neonatal sepsis. However, the systematic research and comparison of these biomarkers for diagnosing NS are limited. For example, we have investigated the diagnosis value of serum amyloid A (SAA) in NS [11]. Here, we continue to evaluate the value of the tumor necrosis factor-*α* (TNF-*α*), by considering it as a useful marker. TNF-*α* is a cytokine involved in systemic inflammation, which belongs to a member of a group of cytokines that stimulate the acute phase reaction [12].

Thus, the objective of this meta-analysis was to investigate the value of TNF-*α* for detecting NS. Although a lot of works indicate that both PCT and CRP are two superior markers for diagnosis of sepsis and infection, we suggest here the TNF-*α* is also a promising marker in NS. A deeper meta-analysis of these studies is thus currently needed.

## 2. Methods

### 2.1. Studies Retrieval and Selection

In order to perform a systematic analysis of the available evidence on the efficacy of TNF-*α* in NS [13], the common approach of literature search was performed in PUBMED, EMBASE, and the Cochrane Library for relevant citations from March 1994 to August 2013. The search terms used were “TNF-*α*,” “neonatal,” “neonate,” “sepsis,” “infant,” “newborn,” and “tumor necrosis factors-*α*”. The reference lists of all known primary and review articles were also searched. No language restriction was used, so that we have examined the references of known articles to fully retrieve the data.

If an article does not include enough data for calculating sensitivity and specificity (2 × 2 table), we asked the corresponding author to provide us with necessary data. If there was no response from the corresponding author, a reminder was sent after one week. If we still cannot achieve the data after this process, the study was excluded from meta-analysis. The selection of articles was performed by two investigators independently to ensure the high accuracy.

### 2.2. Data Extraction

Data collected from the studies included the first author, publication year, diagnostic cut-off point and time, test methods, and sensitivity and specificity data. So the numbers of true-positive, false-positive, false-negative, and true-negative results were extracted for each study. Accuracy data was extracted to construct 2 × 2 table at a specific time. We have requested the information from the authors, if no enough data on the criteria was found in the studies.

### 2.3. Statistical Analysis

We used Meta-Disc 1.4 software and Review Manager 5.0 to perform the statistical analysis [14]. Diagnoses were grouped into two groups according to the time of TNF-*α* test for diagnosis of NS. One group is the time points of TNF-*α* measurement for the diagnosis of early-onset neonatal sepsis (EONS), which were 0–72 h of age; the other group is the time points of TNF-*α* measurement for the diagnosis of late-onset neonatal sepsis (LONS), in which the age of neonates is older than 72 h. We calculated the sensitivity, specificity, diagnostic odds ratio (OR), and corresponding 95% confidence intervals (CI) from each study. We also gained the pooled sensitivity, specificity, and diagnosis OR from each group.

The diagnostic OR expresses how much greater the odds of having sepsis are for newborns who have a positive test result, relatively to newborns who have a negative result [15]. For the estimates of diagnostic OR, heterogeneity was assessed by using the Cochrane *Q* statistic. Normally, *I* ^2^ lies between 0% and 100%. If *I* ^2^ < 50%, then there is a lot of homogeneity among studies in meta-analysis; whereas *I* ^2^ > 50% shows there is more heterogeneity among studies. A value of 0% indicates no observed heterogeneity, and larger values show increasing heterogeneity [16]. We explored the reasons for heterogeneity by carrying out the subgroup analysis and examined characteristics of included studies.

In order to summarize these results, we constructed summary receiver operator characteristic (SROC) curves, which showed the relationship between sensitivity and the false positives (1-specificity). Q* values was received from the SROC curves. Meanwhile, the area under the (SROC) curves was also calculated from the SROC curves, which have been proposed as a way to assess diagnostic data in the context of a meta-analysis [17].

## 3. Results

### 3.1. Study Selection

**Figure 1:**
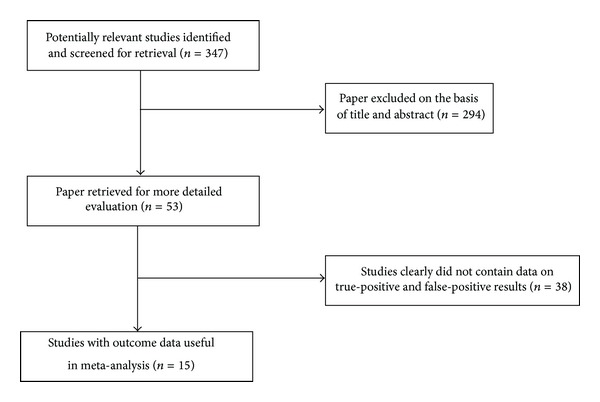
Summary of study assessment and inclusion in the meta-analysis of studies involving diagnosis of neonatal sepsis using a TNF-*α* test.

The literature search was completed in August 2013. We found 347 potentially relevant restudies, but only 15 articles met our inclusion criteria.  Figure 1 shows the chart of literature search. Detailed information for each included study is presented in Table 1.

### 3.2. Accuracy of the TNF-*α* Test in the Diagnosis of Proven Early-Onset Neonatal Sepsis

Eleven articles and twelve trials were included to estimate the use of the TNF-*α* test in the diagnosis of proven early-onset neonatal sepsis (EONS). EONS was defined as the clinical sepsis in the 0–72 h after delivery, met the inclusion criteria in [18–27].

**Figure 2:**
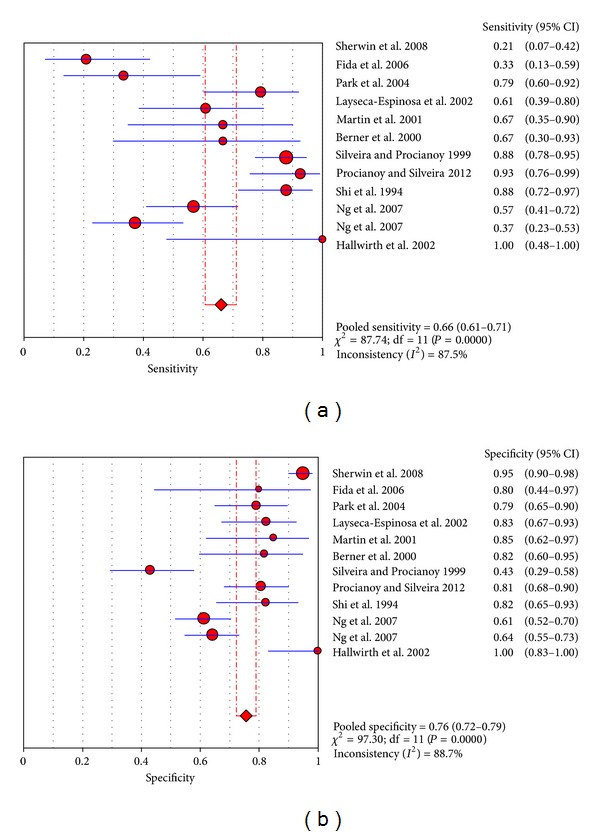
Forest plot for sensitivity and specificity of the TNF-*α* test to diagnose neonatal sepsis at the EONS.

In these trials, we can get the TP, TN, FP, FN, sensitivity, specificity, DOR, PPV, and NPV from the articles. The sensitivity ranged from 20.8% to 100% and pooled sensitivity is 66.1% (95% CI 60.7%–70.1%), specificity ranged from 43.1% to 100% and pooled specificity is 75.6% (95% CI 72.2%–78.9%), and the detailed forest map is shown in Figure 2. We calculated the significant heterogeneity among studies (sensitivity, *I* ^2^ = 87.5%; specificity, *I* ^2^ = 88.7%); it indicated that patient selection or other relevant factors might be responsible for heterogeneity.

**Figure 3:**
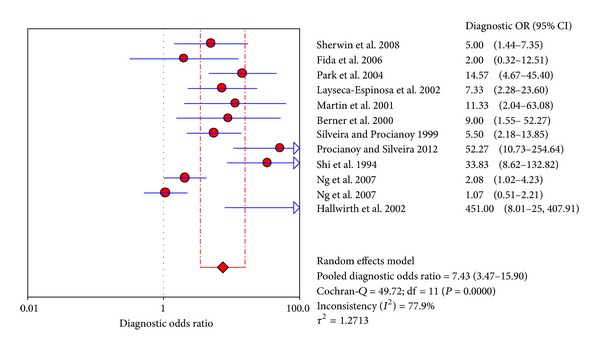
Forest plot for diagnostic OR of the TNF-*α* test to diagnose neonatal sepsis at the EONS.

**Figure 4:**
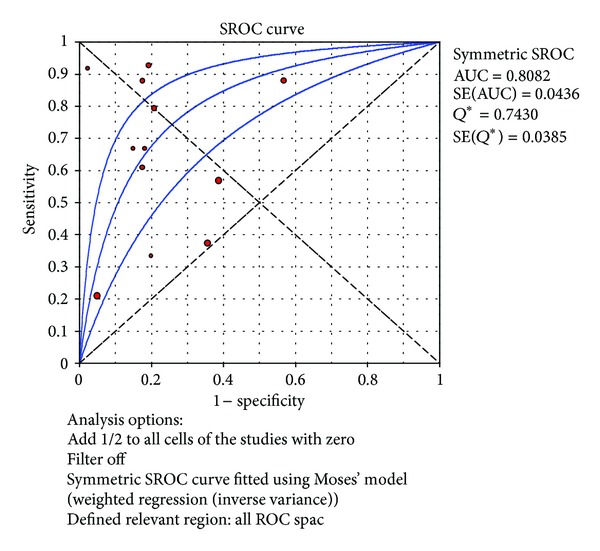
Summary receiver operating characteristic (SROC) curve of the TNF-*α* test for the diagnosis of early-onset neonatal sepsis. Each point represents one study in the SROC curve.

The value of DOR of TNF-*α* was 7.43 (95%CI 3.47–15.90), as shown in Figure 3. In these articles, we calculated the significant heterogeneity (*I* ^2^ = 77.9%). The SROC curve for TNF-*α* markers was plotted in Figure 4; the AUC was 0.81 with the standard error being 0.04. The pooled diagnostic accuracy (Q*) of 0.7430 with the standard error was 0.04.

### 3.3. Accuracy of the TNF-*α* Test in the Diagnosis of Proven Late-Onset Neonatal Sepsis

Six articles and eleven trials were included to estimate the use of the TNF-*α* test in the diagnosis of proven late-onset neonatal sepsis (LONS). LONS was defined as the clinical sepsis 72 h after birth, met the inclusion criteria in [1, 14, 24, 28–30].

**Figure 5:**
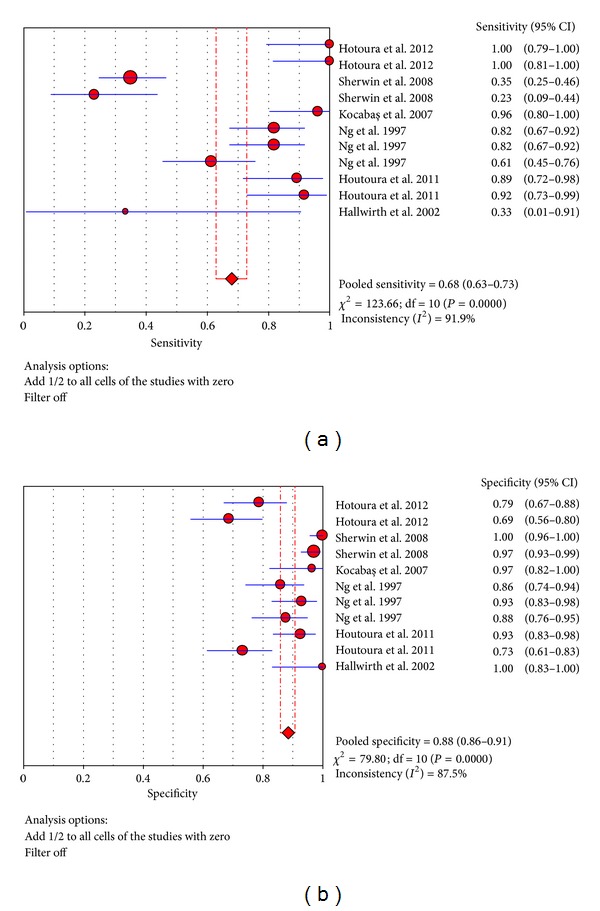
Forest plot for sensitivity and specificity of the TNF-*α* test to diagnose neonatal sepsis at the LONS.

In these trials, we can also get much information from the articles. The sensitivity ranged from 23.1% to 100% and pooled sensitivity is 68.0% (95% CI 62.8%–72.8%), specificity ranged from 73.2% to 100% and pooled specificity is 88.5% (95% CI 85.9%–90.7%), and the detailed forest map is shown in Figure 5. We calculated the significant heterogeneity among studies (sensitivity, *I* ^2^ = 91.9%; specificity, *I* ^2^ = 87.5%), which indicated that patient selection or other relevant factors might be responsible for heterogeneity.

**Figure 6:**
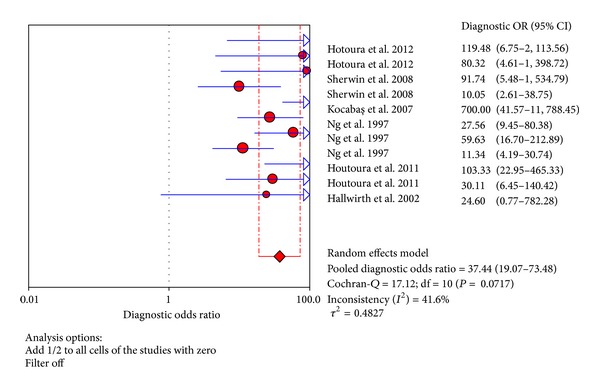
Forest plot for diagnostic OR of the TNF-*α* test to diagnose neonatal sepsis at the LONS.

**Figure 7:**
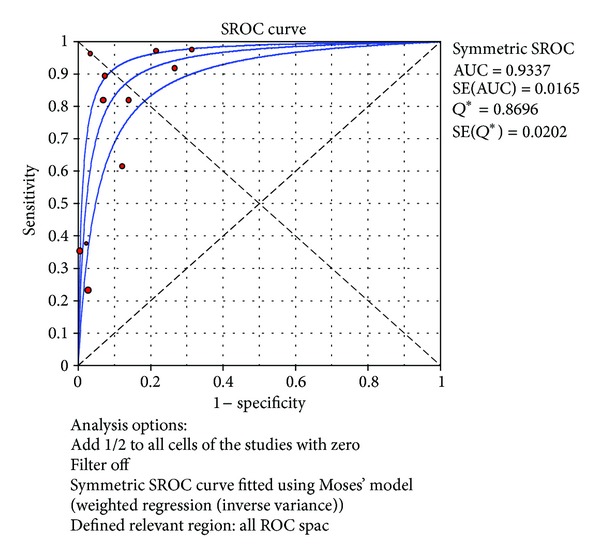
Summary receiver operating characteristic (SROC) curve of the TNF-*α* test for the diagnosis of late-onset neonatal sepsis. Each point represents one study in the SROC curve.

The value of DOR of TNF-*α* was 37.44 (95% CI 19.07–73.48), as shown in Figure 6. In these articles, we calculated the significant heterogeneity (*I* ^2^ = 41.6%). The SROC curves for TNF-*α* markers were plotted in Figure 7; the AUC was 0.93 with the standard error being 0.017. The pooled diagnostic accuracy (Q*) of 0.8696 with the standard error was 0.02.

### 3.4. Intensive Study of the TNF-*α* Test in the Diagnosis of Proven Late-Onset Neonatal Sepsis

In the LONS study, study populations come from different countries, but in general they can be further divided into two regions: the northern hemisphere and the southern hemisphere.

**Figure 8:**
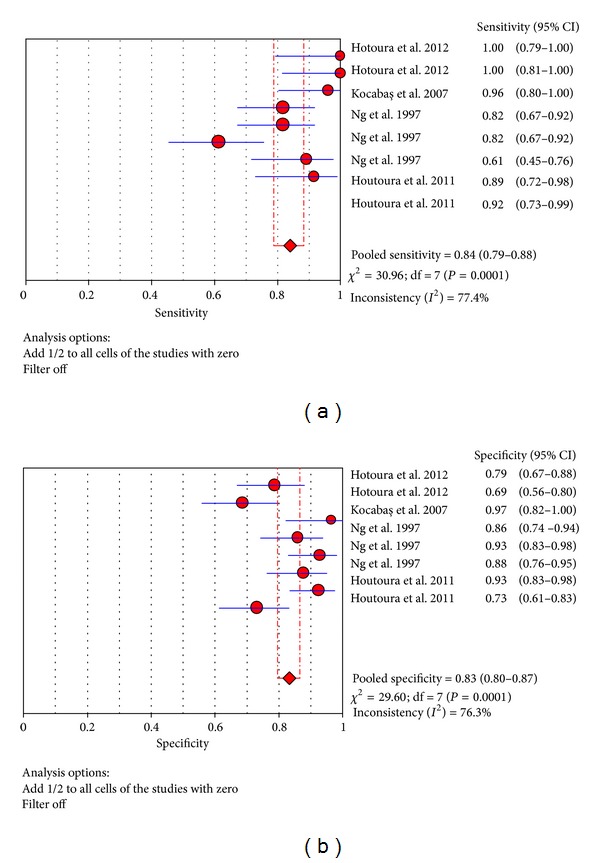
Forest plot for sensitivity and specificity of the TNF-*α* test to diagnose neonatal sepsis at the LONS in the northern hemisphere.

Eight trials were included to estimate the use of the TNF-*α* test in the northern hemisphere at the diagnosis of proven late-onset neonatal sepsis [1, 14, 29, 30]. In these trials, sensitivity ranged from 61.4% to 100% and pooled sensitivity is 84.0% (95% CI 78.8%–88.4%), specificity ranged from 68.8% to 96.6% and pooled specificity is 83.3% (95% CI 79.6%–86.6%), and the detailed forest maps are shown in Figure 8. We calculated the significant heterogeneity among studies (sensitivity, *I* ^2^ = 77.4%; specificity, *I* ^2^ = 76.3%), which indicated that patient selection or other relevant factors might be responsible for heterogeneity.

**Figure 9:**
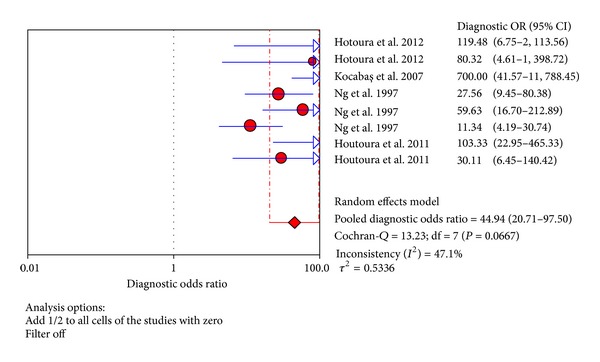
Forest plot for diagnostic OR of the TNF-*α* test to diagnose neonatal sepsis at the LONS in the northern hemisphere.

**Figure 10:**
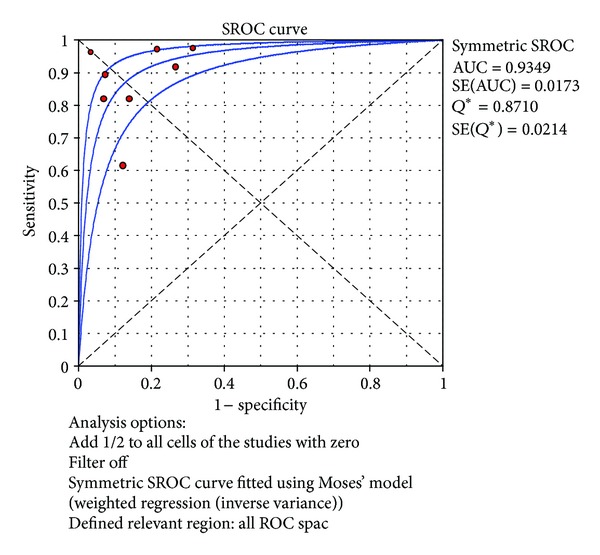
Summary receiver operating characteristic (SROC) curve of the TNF-*α* test for the diagnosis of late-onset neonatal sepsis in the northern hemisphere. Each point represents one study in the SROC curve.

The value of DOR of TNF-*α* test in the northern hemisphere at the diagnosis of proven late-onset neonatal sepsis was 44.94 (95% CI 20.71–97.50), as shown in Figure 9. In these articles, we calculated the significant heterogeneity (*I* ^2^ = 47.1%). The SROC curves for TNF-*α* markers were plotted in Figure 10; the AUC was 0.93 with the standard error being 0.017. The pooled diagnostic accuracy (Q*) of 0.8710 with the standard error was 0.02.

**Figure 11:**
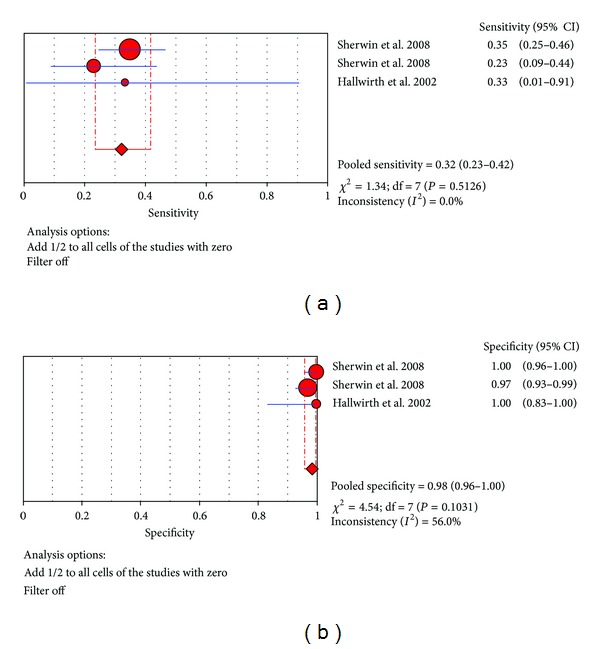
Forest plot for sensitivity and specificity of the TNF-*α* test to diagnose neonatal sepsis at the LONS in the southern hemisphere.

Three trials were included to estimate the use of the TNF-*α* test in the southern hemisphere at the diagnosis of proven late-onset neonatal sepsis [24, 28]. In these trials, sensitivity ranged from 23.1% to 35% and pooled sensitivity is 32.1% (95% CI 23.5%–41.7%), specificity ranged from 97.1% to 100% and pooled specificity is 98.3% (95% CI 95.8%–99.5%), and the detailed forest map is shown in Figure 11. We calculated the significant heterogeneity among studies (sensitivity, *I* ^2^ = 0%; specificity, *I* ^2^ = 56.0%), which indicated that patient selection or other relevant factors might be responsible for heterogeneity.

**Figure 12:**
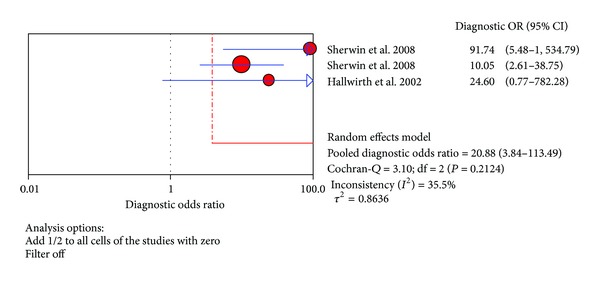
Forest plot for diagnostic OR of the TNF-*α* test to diagnose neonatal sepsis at the LONS in the southern hemisphere.

**Figure 13:**
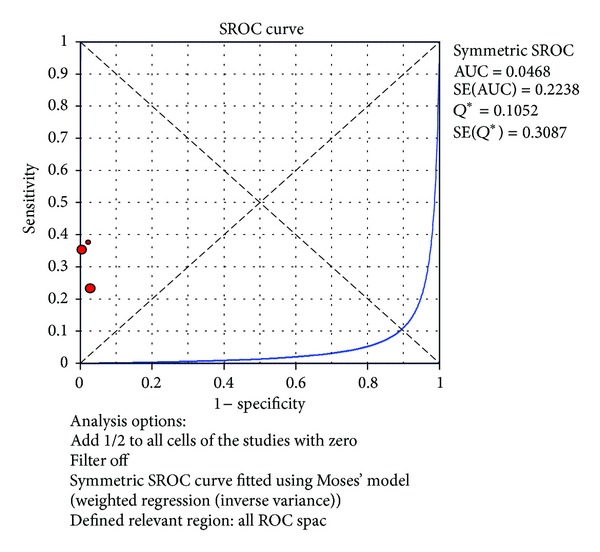
Summary receiver operating characteristic (SROC) curve of the TNF-*α* test for the diagnosis of late-onset neonatal sepsis in the southern hemisphere. Each point represents one study in the SROC curve.

The value of DOR of TNF-*α* test in the northern hemisphere at the diagnosis of proven late-onset neonatal sepsis was 20.88 (95% CI 3.84–113.49), as shown in Figure 12. In these articles, we calculated the significant heterogeneity (*I* ^2^ = 35.5%). The SROC curves for TNF-*α* markers were plotted in Figure 13; the AUC was 0.0468 with the standard error being 0.224. The pooled diagnostic accuracy (Q*) of 0.1052 with the standard error was 0.309.

### 3.5. Comparison of the Diagnostic Accuracy of Markers for Neonatal Sepsis

In LONS, PCT and CRP have been proven to be useful markers for the diagnosis of NS [3, 9, 31]. In order to show the value of diagnosis of the TNF-*α* test for NS in LONS, we compared TNF-*α* with PCT and CRP in LONS. Six articles and eleven trials were used to evaluate the diagnosis of TNF-*α*. Compared with 55% (95% CI 45%–65%) for the CRP test and 72% (95% CI 63%–81%) for the PCT test [14], the pooled sensitivity for the TNF-*α* test was 68% (95% CI 63%–73%).

The pooled specificity for the TNF-*α* was slightly higher than for the CRP and PCT test (88.5% (95% CI 86%–91%) versus 85% (95% CI 81%–88%) versus 77% (95% CI 72%–81%)). Furthermore, the pooled diagnostic OR for the TNF-*α* was higher than CRP and PCT (37.4 (95% CI 19.1–73.5) versus 8.6 (95% CI 3.5–21.0) versus 11.6 (95% CI 5.2–26.0)). The Q* value was slightly higher for the TNF-*α* than CRP and PCT (0.87 versus 0.75 versus 0.77). In SROC curve the TNF-*α*'s AUC is almost equal to CRP (0.93 versus 0.96). As many articles reported that PCT and CRP are good markers, the TNF-*α* is also a good marker for the diagnosis of NS in LONS.

### 3.6. Analysis of Heterogeneity

Heterogeneity is very critical in a meta-analysis, so we should try to explore the reason for the heterogeneity. Generally speaking, variations include several influence factors, for instance the cut-off value, study population, inject antibodies, and so forth.

Firstly, we consider the cut-off value. In our study, many data are so large, so we suppose that this is the possible reason for the heterogeneity. We excluded two studies of [24, 28] whose cut-off values were relatively large. But the *I* ^2^ (heterogeneity test) almost does not change (41.7% versus 41.6%). So the cut-off value is not the reason for the heterogeneity. Secondly, we consider the study population. The *I* ^2^ is reduced from 41.6% to 0% when two studies of [28, 29] were excluded from the meta-analysis. Their study population is different from others. So the study population may be the major reason for the heterogeneity.

### 3.7. Publication Bias

The publication bias is difficult to avoid in meta-analysis [32]. In each study, we often choose favorable results and give up negative results; many “blindness” of test results was never reported. The limit of current available data may bias our conclusion.

## 4. Discussion

NS is one of the most common diseases and life-threating disorder in neonate, and thus it can bring the high mortality and morbidity in infants. So the identification of biomarkers is very important to improve the diagnosis of NS. The clinical signs are nonspecific and laboratory indicators including blood culture are not reliable [33]. The sensitivities of markers are not always so high [34]. So it is necessary to find a good marker for NS.

It is well known that an excellent marker should have high sensitivity and specificity. In our meta-analysis, the TNF-*α* tests' sensitivity is 0.66 for the diagnosis of early-onset neonatal sepsis, and the specificity is 0.46 and the Q* is 0.74. At the late-onset neonatal sepsis, the TNF-*α* test's sensitivity is 0.68, whereas the specificity is 0.89 and the Q* is 0.87. In particular, TNF-*α* shows a higher accuracy for the diagnosis of NS in LONS. Therefore, we have further analyzed the regional issues in LONS. To this end, we have classified studies into two groups, that is, northern hemisphere group [1, 14, 29, 30] and southern hemisphere group [24, 28]. Our analysis found that the northern hemisphere group has higher sensitivity and specificity (sensitivity = 84%, specificity = 83%). The results show that the TNF-*α* has appropriate accuracy for the diagnosis of NS and thus is a good biomarker for the diagnosis of NS.

CRP is an excellent marker and has been applied in clinic [10]. The sensitivity of CRP is 30%–97%, and the specificity ranged from 75% to 100% [35]. In our meta-analysis, TNF-*α*' sensitivity ranged from 23.1% to 100%, and specificity ranged from 73.2% to 100%. The study from TNF-*α* is similar to CRP, so the TNF-*α* is a useful marker in the diagnosis of NS. In addition, PCT is more excellent marker which has better accuracy than CRP for the diagnosis of NS [3]. In our meta-analysis, the pooled sensitivity of TNF-*α* is slightly lower than that of the PCT test in EONS (66.1% versus 74%), the pooled specificity also in such (76% versus 86%). But in LONS, the pooled specificity of TNF-*α* is higher than that of the PCT test (89% versus 77%), although the pooled sensitivity is slightly lower than PCT (68% versus 72%). Generally speaking, the data of TNF-*α* and PCT is greater than CRP. This result again shows that TNF-*α* is good marker in the diagnosis of NS.

In conclusion, TNF-*α* shows the moderate accuracy in the diagnosis of NS, both in EONS and LONS. If we test the accuracy of TNF-*α* by further dividing data into two regions, the study in northern hemisphere shows a better result. Because of the relatively few testing data, the experiments results need to be further studied and the clinical validation is also needed.

## Figures and Tables

**Table 1:** Characteristics of studies included in the meta-analysis of the diagnosis of neonatal sepsis using a TNF-*α* test.

Study	Study population	Patients (*n*)	Sepsis diagnosis	Cut-off (pg/mL)	Time	Country
Hotoura et al. 2012 [1]	Cases: newborns with suspected sepsis Control: infection-free infants	82	Culture; clinical	30	LONS	Greece
Hotoura et al. 2012 [1]	Cases: newborns with suspected sepsis Control: infection-free infants	82	Culture; clinical	15	LONS	Greece
Sherwin et al. 2008 [28]	Cases: NICU newborns with suspected sepsis Control: neonates without sepsis	164	Culture; clinical	180	EONS	New Zealand
Sherwin et al. 2008 [28]	Cases: NICU newborns with suspected sepsis Control: neonates without sepsis	164	Culture; clinical	70	LONS	New Zealand
Sherwin et al. 2008 [28]	Cases: NICU newborns with suspected sepsis Control: neonates without sepsis	164	Culture; clinical	180	LONS	New Zealand
Kocabaş et al. 2007 [14]	Cases: neonates with a suspected clinical sepsis Control: healthy neonates without infectious	55	Culture; clinical	7.5	LONS	Turkey
Fida et al. 2006 [18]	Cases: neonates with clinical or proven or possible infected sepsis Control: disease without infection	28	Culture; clinical	29.86	EONS	Saudi Arabia
Park et al. 2004 [19]	Cases: newborns with suspected sepsis Control: neonates without sepsis	77	Culture; clinical	41	EONS	Korea
Layseca-Espinosa et al. 2002 [20]	Cases: neonates with clinical or proven sepsis Control: disease without infection	63	Culture; clinical	0.18	EONS	Spain
Martin et al. 2001 [21]	Cases: newborns with suspected sepsis Control: neonates without sepsis	32	Culture; clinical	20	EONS	Sweden
Berner et al. 2000 [22]	Cases: newborns with suspected sepsis Control: neonates without sepsis	31	Culture; clinical	48	EONS	Germany
Silveira and Procianoy 1999 [23]	Cases: newborn infants with clinical sepsis or probably infected with clinical sepsis Control: neonates without sepsis	117	Culture; clinical	12	EONS	Brazil
Ng et al. 1997 [29]	Cases: VLBW infants with suspected clinical sepsis Control: noninfected newborns	101	Culture; clinical	17	LONS	Hong Kong
Ng et al. 1997 [29]	Cases:VLBW infants with suspected clinical sepsis Control: noninfected newborns	101	Culture; clinical	17	LONS	Hong Kong
Ng et al. 1997 [29]	Cases: VLBW infants with suspected clinical sepsis Control: noninfected newborns	101	Culture; clinical	17	LONS	Hong Kong
Hotoura et al. 2011 [30]	Cases: full-term neonates with suspected or documented infection Control: infection-free infants	95	Culture; clinical	30	LONS	Greece
Hotoura et al. 2011 [30]	Cases: full-term neonates with suspected or documented infection Control: infection-free infants	95	Culture; clinical	15	LONS	Greece
Hallwirth et al. 2002 [24]	Cases: neonates with sepsis Control: neonates without sepsis	25	Culture; clinical	20000	LONS	Austria
Procianoy and Silveira 2012 [25]	Cases: very low birth weight infants with clinical sepsis Control: neonates without sepsis	84	Culture; clinical	30	EONS	Brazil
Shi et al. 1994 [26]	Cases: neonates with sepsis Control: neonates without sepsis	67	Culture; clinical	267.2	EONS	CHINA
Ng et al. 2007 [27]	Cases: very low birth weight infants with suspected sepsis Control: neonates without sepsis	155	Culture; clinical	0.6	EONS	Hong Kong
Ng et al. 2007 [27]	Cases: very low birth weight infants with suspected sepsis Control: neonates without sepsis	155	Culture; clinical	0.6	EONS	Hong Kong
Hallwirth et al. 2002 [24]	Cases: neonates with sepsis Control: neonates without sepsis	25	Culture; clinical	20000	EONS	Austria
